# Chromosome interaction over a distance in meiosis

**DOI:** 10.1098/rsos.150029

**Published:** 2015-02-25

**Authors:** Mary Brady, Leocadia V. Paliulis

**Affiliations:** Biology Department, Bucknell University, Lewisburg, PA 17837, USA

**Keywords:** meiosis, chromosome movements, distance segregation, cell division

## Abstract

The challenge of cell division is to distribute partner chromosomes (pairs of homologues, pairs of sex chromosomes or pairs of sister chromatids) correctly, one into each daughter cell. In the ‘standard’ meiosis, this problem is solved by linking partners together via a chiasma and/or sister chromatid cohesion, and then separating the linked partners from one another in anaphase; thus, the partners are kept track of, and correctly distributed. Many organisms, however, properly separate chromosomes in the absence of any obvious physical connection, and movements of unconnected partner chromosomes are coordinated at a distance. Meiotic distance interactions happen in many different ways and in different types of organisms. In this review, we discuss several different known types of distance segregation and propose possible explanations for non-random segregation of distance-segregating chromosomes.

## 2. Introduction

**Figure 1.**
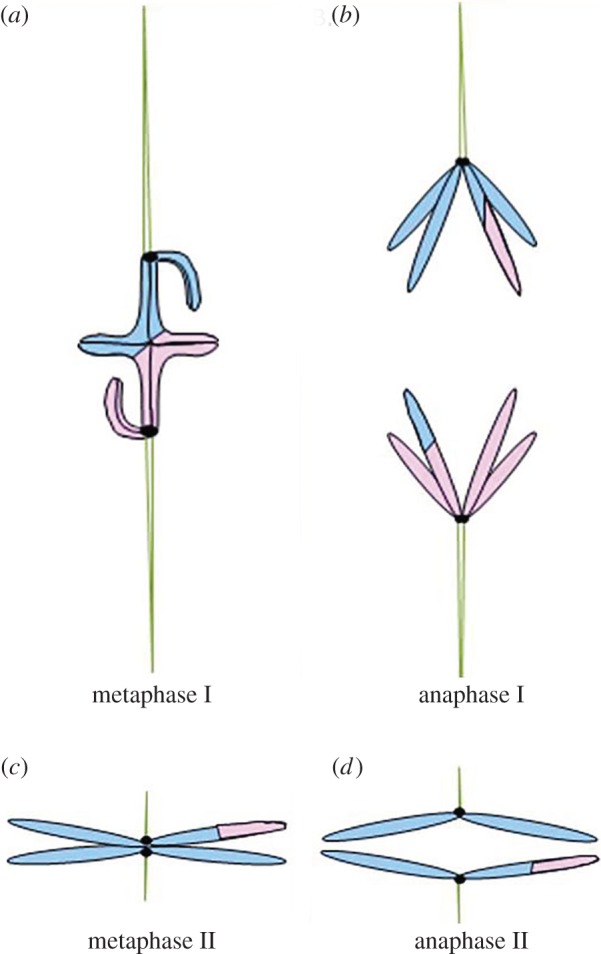
Behaviour of metacentric chromosome in ‘standard’ meiosis. In meiosis I, each bivalent has two pairs of sister chromatids; each pair of sisters constitutes one homologue. The homologues are held together, because recombination forms a connection, called a chiasma, between the two. (*a*) The site of recombination (arrowhead) is represented as a change in colour (pink and blue interchange) on the chromosomes. (*b*) Homologues separate in anaphase I, and the cell enters meiosis II. (*c*) In metaphase II, sister kinetochores associate with opposite poles. (*d*) In anaphase II, sister chromatids separate from one another, with each gamete getting a single copy of each chromosome.

Meiosis produces haploid gametes from a diploid parent cell that has undergone DNA replication. To produce viable, healthy and fertile offspring, chromosomes must be segregated correctly into gametes such that each gamete has a single copy of each chromosome. Correct segregation is guaranteed by a highly regulated series of events. In ‘standard’ meiosis, DNA replication occurs; each autosome and sex chromosome then finds its partner in the nucleus during prophase I. Homologues and even heterologous sex chromosome can then undergo recombination in regions of sequence homology. The visible evidence of this recombination or crossing over is called a chiasma. Physically connected partner chromosomes form a bivalent that attaches to the spindle; one partner associates with one pole, and the other partner associates with the opposite pole. Each partner comprises a pair of sister chromatids, and sister kinetochores face the same spindle pole during meiosis I (figure 1*a*). Partners, either homologues or heterologous sex chromosomes, then separate from one another in anaphase I (figure 1*b*). After a brief resting phase (interkinesis), replicated sister chromatids, held together by cohesion between sister centromeres, attach to newly formed spindles (figure 1*c*). In meiosis II, sister kinetochores face opposite spindle poles; in anaphase II, the sister chromatids separate (figure 1*d*) [1,2].

Meiosis happens properly because of chromosome geometry (figure 1). The connection established between homologous chromosomes during recombination causes a bivalent to form and allows for proper positioning of partner chromosomes on the meiosis I spindle (figure 1*a*,*b*). This physical connection is a sort of communication between the homologues that allows each chromosome to monitor the position of its partner. Additionally, a meiotic cell only receives appropriate signals from a chromosome if it is built properly, consequently kinetochore-associated tension can contribute to signalling and complete attachment of a chromosome to the spindle. In the absence of correct chromosome construction and recombination, most meiotic cells produce aneuploid gametes [1–3].

However, not all meiotic chromosomes require a physical connection with their partner chromosome to become properly positioned on a meiotic spindle. Coordination between and accurate separation of partner chromosomes can happen in the absence of a chiasma or any other obvious physical connection.

Hughes-Schrader [4] coined the term distance segregation to describe the process by which apparently *unconnected* partner chromosomes always separate from one another. Distance segregation is an excellent system for studying how the position of meiotic chromosome is communicated and regulated within meiotic cells because unconnected partner chromosome movements are so visibly and so carefully regulated. Based on cytological observations distance segregation systems vary significantly, but in each such system at least one pair of autosomes or sex chromosomes are unconnected. Amazingly, the partner chromosomes always manage to segregate properly either early or late in anaphase in the absence of visible connection. Forms and variations of distance segregation have been observed in bryophytes, insects and flatworms [4–12]. The process of meiosis itself (‘standard’ or atypical) has only been closely studied in a small number of systems, and there is considerable variation among these few systems; therefore, distance segregation probably occurs in other taxa.

## 3. Distance segregation is highly variable

**Figure 2.**
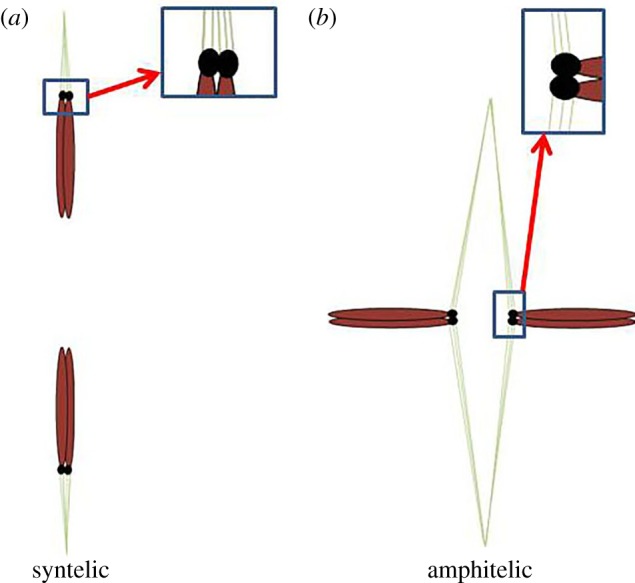
Types of chromosome orientation. (*a*) In chromosomes with a syntelic attachment to the spindle, both sister kinetochores face and associate with microtubules coming from the same spindle pole (inset, shown by red arrow). (*b*) In chromosomes with amphitelic attachment to the spindle, sister kinetochores face and associate with microtubules coming from opposite spindle poles (inset, shown by red arrow).

In all known distance-segregating systems, one or more pairs of chromosomes correctly segregate in the absence of a visible physical connection and in an environment in which typical chromosome pairs also exist and segregate correctly. In meiosis I, distance segregation is a special case of achiasmate or distributive segregation. There are cases of achiasmate segregation in which partner chromosomes are connected to one another—e.g. homologues are connected, but achiasmate in *Drosophila* primary spermatocytes [13]. Most distance-segregating chromosomes observed to date are heterologous sex chromosomes, and distance segregation has been most closely studied in male meiosis I. Nevertheless, there are examples of distance segregation within the flatworm genus *Mesostoma*, several species of liverworts, and in the insect order Hemiptera that involve segregation of pairs of univalent autosomes (or what appear to be autosomes, as there is no difference in size between the two partners). Timing of segregation of partner chromosomes in distance-segregating systems is variable. In some systems, partners are separate prior to nuclear envelope breakdown [4,10,14]. In other systems, the distance-segregating univalents move to opposite poles long after all the bivalents in the cell separate [7,15]. Moreover, distance-segregating chromosomes are highly variable in how they attach to the spindle. In some cases, each partner in a distance-segregating pair forms a syntelic attachment to the spindle, i.e. both sister kinetochores face the same pole (figure 2*a*). In other cases, each partner forms amphitelic attachments to the spindle, i.e. the sister kinetochores face opposite poles (figure 2*b*). Notably, distance-segregating systems can have either syntelic or amphitelic chromosome attachments in distance-segregating chromosomes. Distance segregation has been observed not only in plants (liverworts) and animals (flat worms and several orders of insects), but may also exist in other taxa. Distance segregation is typically observed in spermatocytes for two major reasons. First, spermatocyte meiosis is more easily studied cytologically than oocyte meiosis. Second, distance-segregating chromosomes are usually heteromorphic sex chromosomes. Further study might uncover clear-cut cases of distance segregation in females. Additionally, distance segregation is typically observed in meiosis I, but distance segregation also occurs in meiosis II.

Here, we review cytological observations from distance-segregating systems and elaborate on the various types of distance-segregation systems with a focus on the timing of separation of partner chromosomes. We discuss findings from micromanipulation, microbeam-irradiation and laser-ablation experiments. In addition we discuss how chromosome behaviour in *Drosophila* female meiosis could inform our current understanding of distance segregation.

## 4. Partner sex chromosomes can segregate in prometaphase, prior to autosome congression to spindle equator

**Figure 3.**
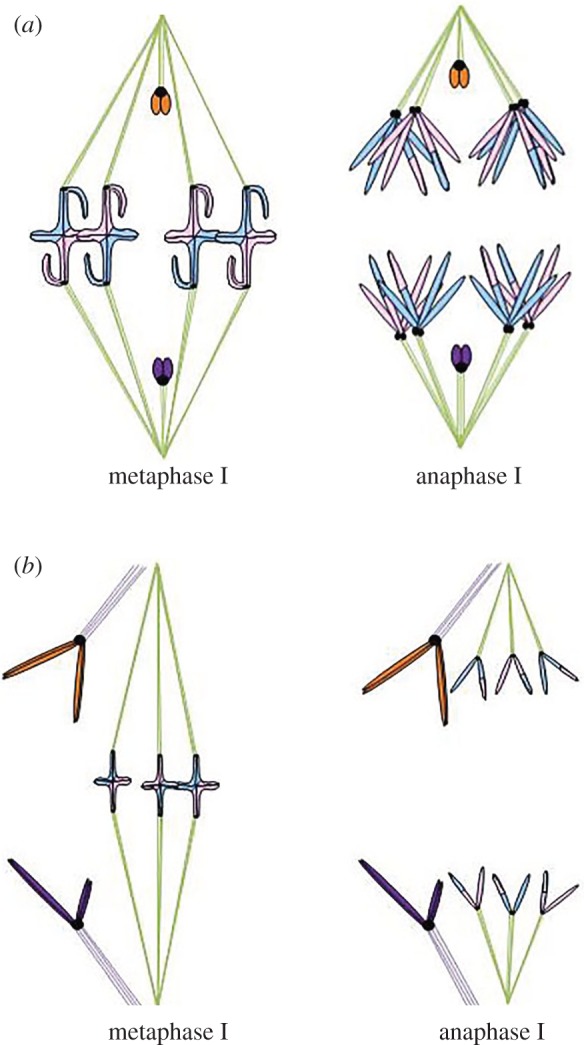
Separation of X and Y chromosomes in or before autosomal metaphase I. In spermatocytes of many organisms, either unpaired sex chromosomes or unpaired univalent chromosomes are not connected in meiosis I. (*a*) In metaphase, the unpaired chromosomes associate closely with opposite spindle poles (purple and orange chromosomes), whereas the autosomes (pink and blue chromosomes, with colour changes showing the position of chiasmata) line up on the metaphase plate. In anaphase I, all chromosomes move towards their associated spindle pole. (*b*) In organisms with a separate sex spindle (i.e. some flea beetles), unpaired chromosomes associate closely with opposite spindle poles of the sex spindle (purple and orange chromosomes), whereas the autosomes line up on the metaphase plate of the autosomal spindle. In anaphase I, all chromosomes move towards their associated spindle pole.

In spermatocytes of some plant and many insect species, the condensed X and Y sex chromosomes are not physically paired in metaphase I; each sex chromosome emerges from meiotic prophase I as a univalent, and the segregation partners remain separate throughout metaphase I. In liverwort species with XX–XY sex determination that display distance segregation, the univalent X and Y chromosomes each attach syntelically to spindles in male meiotic tissues; they then separate from each other without any apparent prior connection [5]. Similar chromosome behaviour is observed in the insect orders Neuroptera and Raphidioptera, which have univalent X and Y chromosomes that separate in or before metaphase (figure 3*a*). Most species in these orders have a single X and a single Y chromosome. The X and Y chromosomes appear to be enveloped in a separate unit of the spindle that is located in the centre of the spindle, whereas the autosomes are located more peripherally near the spindle edges [4,8–10,16–18]. In some Neuroptera (the Mantispidae), some of the sex chromosome pairs are linked in prophase I, but they separate in metaphase I, well before anaphase I [9]. In all of these species, the sex univalents separate from their partners without having a visible connection to that partner.

Like some Neuroptera, some flea beetles (order Coleoptera) place their univalent sex chromosomes in a separate spindle unit. However, in these cases, the unit is peripheral to the spindle unit that contains the autosomes (figure 3*b*). While each autosome pair appears as a bivalent in metaphase I, the univalent sex chromosomes on the peripheral spindle unit are separate throughout metaphase I. In *Alagoasa* (*Oedonychus*) *bicolor* and *Omophoita albicollis*, sister centromeres orient syntelically and the giant, asynaptic sex chromosomes form a ‘distance bivalent’ that is always well separated from the autosomal bivalents on the meiosis I spindle [14]. This pair of sex chromosomes appears to be excluded from the autosomal spindle [19]. Virkki [14] suggested that the sex chromosomes were placed on a separate sex spindle, because the spindle region where sex chromosomes were located was apparently physically separated from the main spindle by a thick layer of cytoplasm and mitochondria [14,19]. Moreover, in disrupted cells, the sex chromosomes and associated spindle fibres moved as a separate unit away from the autosomes and their associated spindle fibres as the cytoplasm flowed away from the plasma membrane [14]. Forer & Wilson [20] used immunofluorescence to label microtubules and confirmed that the sex chromosomes associate with a spindle unit that is separate from the main spindle that associates with autosomes. These results indicate that the X and Y chromosomes in *Alagoasa bicolor*, as observed in some Neuroptera and Raphidioptera species, segregate on a separate spindle unit. The location of that unit differs among species, with *Alagoasa* and other related flea beetles having the separate sex spindle unit peripheral to the autosomal spindle, and Neuroptera and Raphidioptera having the sex spindle unit at the centre of the spindle. The X and Y chromosomes remain separate from one another throughout metaphase I and early anaphase I, they then separate further or move further poleward, when spindle poles separate in late anaphase I [20].

## 5. Distance segregation of multiple sex chromosomes

**Figure 4.**
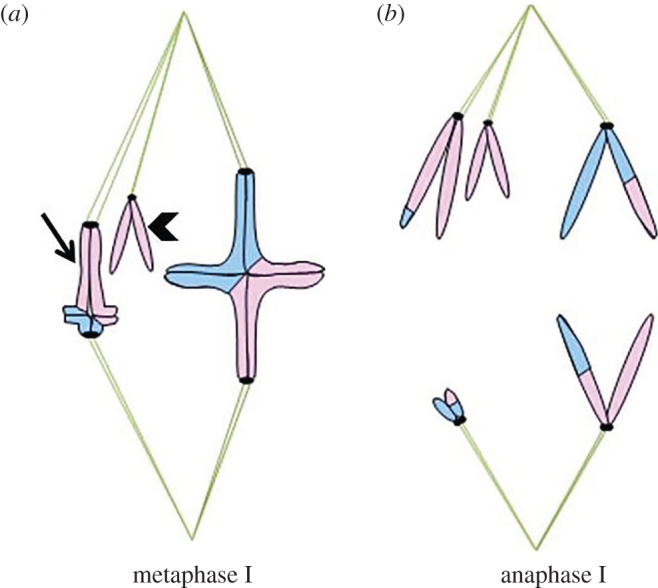
Distance segregation in a distance sex trivalent. In spermatocytes of the mole cricket *Neocurtilla hexadactyla*, males form an X_2_–Y bivalent (arrow) and an X_1_ univalent (arrowhead). (*a*) In metaphase I, X_1_ and X_2_ associate with the same spindle half, though the X_1_ and X_2_ chromosomes are not physically connected. (*b*) In anaphase I, X_1_ and X_2_ move together towards one spindle pole, whereas the Y chromosome moves towards the opposite spindle pole.

The sex chromosomes of the northern mole cricket *Neocurtilla hexadactyla* show that organisms with multiple sex chromosomes can display distance segregation. *N. hexadactyla* females are X_1_X_1_X_2_X_2_, whereas males are X_1_X_2_Y. Males of this species form a heteromorphic X_2_–Y bivalent during prophase I, whereas X_1_ remains a univalent separate from the X_2_–Y bivalent. In metaphase I of male meiosis, the X_2_–Y bivalent aligns on the metaphase plate, and the X_1_ univalent associates with the same spindle pole as the X_2_ half-bivalent (figure 4*a*). In anaphase I of male meiosis, X_1_ and X_2_ move together towards one spindle pole, so that female-forming sperm will result; simultaneously, the Y chromosome moves towards the opposite pole, so that male-forming sperm will result (figure 4*b*) [6,21]. Micromanipulation experiments confirmed the idea that the sex chromosomes undergo non-random segregation with the unconnected X_1_ and X_2_ chromosomes segregating to the same pole. When the X_1_ univalent orientation was reversed, the X_2_Y bivalent did not reorient in any cases, which would lead to formation of aneuploid gametes. However, when orientation of the X_2_Y bivalent was reversed, the X_1_ univalent reoriented on the spindle to associate with the same pole as the X_2_ kinetochore, and all resulting gametes would be euploid [6]. Irradiations of spermatocyte spindle fibres were conducted with monochromatic ultraviolet light [21]. Results of these experiments show that X_1_ must be the active chromosome that is responsible for this non-random segregation. A spindle fibre network may form between X_1_ and the X_2_Y bivalent [21,22]. Upon irradiation of its own spindle fibre or any spindle fibre associated with the X_2_Y bivalent, the X_1_ univalent reorients after irradiation. However, X_1_ does not move or reorient when autosomal spindle fibres are irradiated. Effects of spindle fibre irradiation differ between univalents on the spindle equator and those off the equator; specifically, only univalents that were off the equator underwent reorientation [21].

The non-random segregation in *Neocurtilla* could be due to connections between the X_1_ and Y kinetochores, because electron microscopy indicates that a few microtubules extend between the two chromosomes. Alternatively, it could be due to the presence of electron-dense material associated with the X_1_ and Y kinetochore regions [22]. However, whether the electron-dense material or connections between kinetochores actually facilitate non-random segregation is not yet known.

## 6. Partner sex chromosomes can segregate after autosomal partners segregate

**Figure 5.**
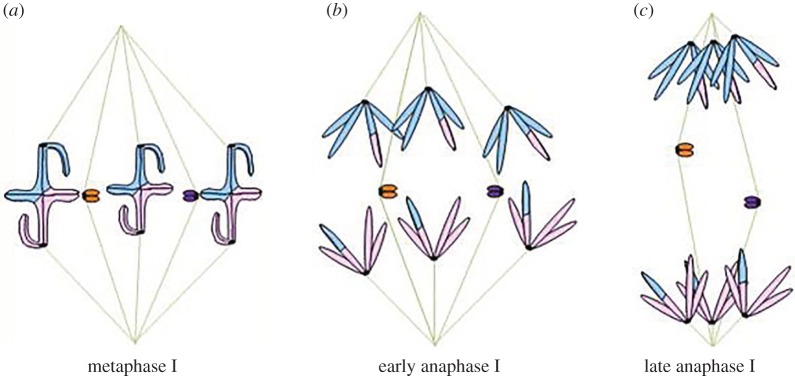
Late anaphase separation of univalent X and Y chromosomes. In primary spermatocytes of crane flies, fungus gnats and some flea beetles, unpaired sex chromosomes (purple and orange chromosomes) align on the metaphase plate with autosomes (pink and blue chromosomes, with colour changes showing the position of chiasmata) (*a*). After autosomes separate and move towards their associated spindle poles (*b*), sex chromosomes initiate movement, one towards each pole (*c*).

Crane flies, fungus gnats and flea beetles of the tribe Alticini (which is different from the flea beetle tribes described above) have sex chromosomes that display a very different form of distance segregation from that described above. Crane flies, fungus gnats and some flea beetles have XX–XY sex determination, and the X and Y chromosomes are univalents, as in Neuroptera and Raphidioptera. However, unlike Neuroptera or Raphidioptera, during metaphase I all chromosomes (including each bivalent autosomal pair and each sex univalent) line up at the equator (figure 5*a*). The autosomes then segregate normally to the poles in anaphase, but the sex chromosomes remain at the equator as autosomes segregate to their associated spindle poles (figure 5*b*). Approximately 25–40 min after the beginning of autosomal chromosome segregation in anaphase, each sex chromosome begins moving towards a pole, and almost simultaneously they segregate to opposite poles (figure 5*c*) [15,23,24].

Behaviour of the distance-segregating X and Y chromosomes has been studied extensively in crane fly spermatocytes. Each sex chromosome, the X and the Y, has amphitelic attachment; spindle fibres that are detectable by birefringence attach each sex chromosome to both poles [7]. Univalent sex chromosomes maintain amphitelic attachment even after they begin moving towards one spindle pole, with one kinetochore fibre shortening and the other lengthening (figure 5, late anaphase I). Their poleward movement is slower than autosomal chromosome movement [25], and it is thought that the lengthening kinetochore fibre slows poleward movement of the respective sex univalent during anaphase [26,27].

Micromanipulation experiments have been performed in early anaphase I, soon after the X and Y chromosomes initiate poleward movement and after all of the autosomes have segregated towards the poles [28]. When one sex chromosome is pushed towards one spindle pole, the other sex chromosome moves towards the opposite pole in response. In laser-irradiation experiments, when a laser is used to separate sister chromatids, each sister kinetochore moves towards the spindle pole it faces. This result shows that the amphitelic attachment is maintained through anaphase I, and that each kinetochore maintains a functional attachment to the respective spindle pole [27]. Many interesting mysteries remain; for example, the signal which determines which kinetochore fibre of each univalent will elongate, and thus which direction each sex univalent will move, is still unknown.

## 7. Evidence that autosomes can also exhibit distance segregation

Autosomes have also been shown to undergo distance segregation in some liverwort species [5] and in some bugs (Hemiptera, Coreidae) [5]. In both cases, the M-chromosomes, which are small autosomes, separate before the larger autosomes separate. In liverworts, the M-chromosomes appear separate within the prophase I nucleus and maintain separation through anaphase I [5]. In some bug species, the M-chromosomes are separate in prophase I, move together and touch on the spindle in metaphase, and then separate during anaphase, but do so before the larger autosomes [29].

**Figure 6.**
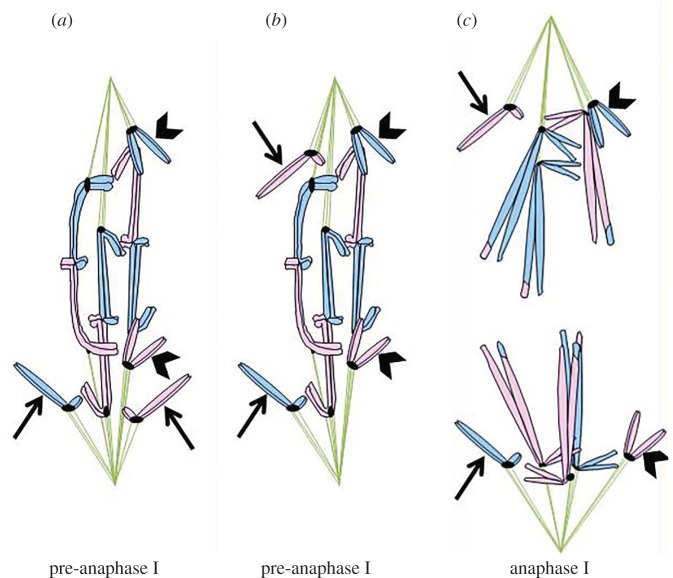
Movement of putative distance-segregating autosomes in *Mesostoma ehrenbergii*. In primary spermatocytes of the flatworm *M. ehrenbergii*, there are three bivalents and two pairs of univalents (one univalent pair shown by an arrow, the other by an arrowhead). (*a*) Prior to anaphase I, the bivalents have bipolar attachments to the spindle, and the univalents (arrow, arrowhead) associate with one spindle pole at a time; sometimes incorrectly—with homologous univalents associating with the same pole (arrows). (*b*) Oscillations and reorientation can happen multiple times prior to anaphase I. Reorientation leads to a correct arrangement of univalents, with one homologous univalent associating with each spindle pole (arrows). (*c*) In anaphase I, half-bivalents and univalentsI (arrow, arrowhead) move towards the pole with which they have associated.

The best-studied example of distance segregation of apparent autosomes (i.e. partners appear to be the same size, and there is no visible difference between chromosome complement in oocytes and spermatocytes) comes from the flatworm *Mesostoma ehrenbergii* [30]. In meiosis I of these flatworms, three homologous pairs of chromosomes form bivalents (chromosomes 1, 3 and 4), and two homologous pairs do not form bivalents, but remain univalent (chromosomes 2 and 5; figure 5). Following nuclear envelope breakdown of meiosis I, each bivalent establishes bipolar attachment to the spindle and then oscillates between the spindle poles. A metaphase plate never forms, because each bivalent continues to oscillate, and sometimes reorients. At the same time, univalent pairs associate with the spindle and each univalent frequently shifts position from being near one spindle pole to being near the other (figure 6*a*) [11,30–34]. Each univalent makes spindle attachments to only one pole at a time; if a univalent changes direction, it does so by making a new attachment to the opposite pole (figure 6*a*,*b*) [11]. In some cases, a univalent can be observed switching poles after correct segregation had been achieved, or a bivalent might flip, so each kinetochore faces an opposite pole. Reorientation after apparently correct attachment is achieved (i.e. with each bivalent having a bipolar attachment and each pole associated with one of each type of univalent) suggests that *Mesostoma* might have non-random segregation of chromosomes. There may be both coordinated positioning of partner chromosomes, and coordinated positioning of non-partner chromosomes [11,34]. Additionally, univalents and half-bivalents prior to anaphase I will move away from and then back to the same pole, apparently detaching from the pole with which they were associated and then reattaching to that same pole [11,30]. Anaphase movements (figure 6*c*) start in the middle of oscillations, with no apparent stable metaphase prior to anaphase I [30,34]. The trigger that leads to onset of anaphase I is unknown, but it seems to be associated with a euploid distribution of chromosomes.

## 8. Distance segregation in meiosis II

Distance segregation also occurs during meiosis II in some insects, including the Hemiptera, Heteroptera. Bugs of the genus *Nabis* display distance segregation of X and Y chromosomes in male meiosis II. As observed in fixed, stained meiosis I spermatocytes, the X and Y chromosomes are achiasmate [35]. The heterologous sex chromosomes undergo equational division in meiosis I. They behave like mitotic chromosomes in that sister chromatids separate in anaphase, and the heterologous sex chromosomes do not separate from one another. By contrast, the autosomes appear to undergo reductional division in meiosis I [35]. In meiosis II, the autosomal sister chromatids separate, as in standard meiosis, but the X and Y chromosomes are separate and associated with opposite spindle poles from metaphase II through anaphase II [35].

## 9. Hints on the mechanism of distance segregation from *Drosophila*

Images of squashed and stained meiosis I oocytes appear to show that distance segregation may occur in female meiosis in *Drosophila melanogaster* [36]. Chromosome 4 is very small and heterochromatic, and the homologues are not joined by a chiasma in meiosis I [36]. The two achiasmate chromosome 4 univalents are clearly separated, one at each pole, in squash preparations of *Drosophila* oocytes [36]. More recent high-resolution microscopy from Gilliland *et al.* [37] show that the separated chromosome 4 univalents rejoin the main chromosome mass, and that each chromosome takes a defined position on the spindle by metaphase I [37]. Gilliland *et al.* [37] also studied the distribution of non-exchange X chromosomes, in which one of the X chromosomes had multiple nested inversions and thus could not recombine with its homologue; a non-inverted X chromosome. Interestingly, though the chromosome 4 and X chromosome univalents are achiasmate, in some cases, there appear to be fine heterochromatic threads connecting these homologues before they finally align at metaphase [37,38]. The function of these threads remains unknown; they could act as force generators that reel in separate chromosome masses, as a difficult-to-see, but tension-carrying parts of the chromosomes, or as a sort of communicator of positional information. Alternatively, the threads could have some unknown function, unrelated to chromosome segregation. Laser-ablation experiments have shown that elastic, but as-yet invisible tethers can exert a backward force on non-distance-segregating anaphase autosomes in crane fly primary spermatocytes [39]. The existence of these force-transducing tethers suggests a potential role for the sometimes-observed heterochromatic threads in *Drosophila* primary oocytes, though, again, their importance and role remain a mystery. If the fine threads that, in some cases, appear to connect achiasmate chromosomes in *Drosophila* oocytes actually reflect a true physical connection, then *Drosophila* primary oocytes, by definition, do not have distance-segregating chromosomes. However, the interesting behaviour of the achiasmate chromosomes in *Drosophila* primary oocytes may inform our understanding of distance segregation, and molecules that contribute to correct segregation of achiasmate chromosomes in *Drosophila* primary oocytes may also contribute to correct segregation of distance-segregating chromosomes in other systems.

The Nod kinesin-like protein has been implicated in the congression of achiasmate chromosomes to the metaphase plate. Achiasmate chromosomes lacking Nod move precociously to the spindle poles; some of these are ejected from the spindle, and never return to the chromosome mass at metaphase [40,41]. Nod is a non-motile kinesin that drives chromosome movement towards the metaphase plate during cell division [42].

Chromosome behaviour in *Drosophila* oocytes may offer explanations for how distance segregation is regulated in other systems. In the liverworts, Neuroptera, Raphidioptera and some flea beetles, the sex chromosomes, like chromosome 4 homologues in *Drosophila*, have been observed near the spindle pole in metaphase. Nod orthologues in these species may be essential for maintaining chromosome position on the spindle in these other organisms. Notably, distance-segregating chromosomes of liverworts, Neuroptera and Raphidioptera have only been observed in fixed, stained specimens and ultimate congression of the distance-segregating chromosomes to the metaphase plate may occur in liverworts, Neuroptera and Raphidioptera, as observed in *Drosophila* oocytes. In addition, thin chromatin tethers, like those observed in *Drosophila* oocytes, other protein tethers, or some combination thereof could connect the distance-segregating chromosomes in these other species. Live-cell experiments with micromanipulation could be used to test whether such connections exist.

Chromosomes can interact with one another over long distances during meiosis. While distance interactions have been observed in many species, many questions remain regarding why organisms vary so much in positioning these separated chromosomes in meiosis. In addition, the mechanisms responsible for these interactions are largely unknown, and the molecules that control these interactions are unidentified. The combination of available molecular tools, small molecule inhibitors and microscopy makes this an exciting time to study distance chromosome interactions.
